# A Putative Effector CcSp84 of *Cytospora chrysosperma* Localizes to the Plant Nucleus to Trigger Plant Immunity

**DOI:** 10.3390/ijms23031614

**Published:** 2022-01-30

**Authors:** Zhiye Xu, Dianguang Xiong, Zhu Han, Chengming Tian

**Affiliations:** 1 Beijing Key Laboratory for Forest Pest Control, Beijing Forestry University, Beijing 100083, China; xuzhiye987@163.com (Z.X.); hanandzhu@163.com (Z.H.); 2 The Key Laboratory for Silviculture and Conservation of Ministry of Education, College of Forestry, Beijing Forestry University, Beijing 100083, China

**Keywords:** *Cytospora chrysosperma*, effector, pathogenicity, subcellular localization, plant immunity

## Abstract

*Cytospora chrysosperma* is the main causal agent of poplar canker disease in China, especially in some areas with poor site conditions. Pathogens secrete a large number of effectors to interfere the plant immunity and promote their infection and colonization. Nevertheless, the roles of effectors in *C. chrysosperma* remain poorly understood. In this study, we identified and functionally characterized a candidate effector CcSp84 from *C. chrysosperma*, which contained a nuclear localization signal motif at the C-terminal and was highly induced during infection stages. Transient expression of CcSp84 in *Nicotiana benthamiana* leaves could trigger cell death. Additionally, deletion of CcSp84 significantly reduced fungal virulence to the polar twigs, while no obvious defects were observed in fungal growth and sensitivity to H_2_O_2_. Confocal microscopy revealed that CcSp84 labeled with a green fluorescent protein (GFP) was mainly accumulated in the plant nucleus. Further analysis revealed that the plant nucleus localization of CcSp84 was necessary to trigger plant immune responses, including ROS accumulation, callose deposition, and induced expression of jasmonic acid and ethylene defense-related genes. Collectively, our results suggest that CcSp84 is a virulence-related effector, and plant nucleus localization is required for its functions.

## 1. Introduction

*Cytospora chrysosperma* is a necrotrophic fungal pathogen that causes stem canker in a broad range of hosts [1,2]. In nature, the spores of *C. chrysosperma* germinate and produce the hyphae to invade the host plants with poor conditions through the wound. The hyphae then infect the phloem and primary xylem in the horizontal and vertical directions and finally extend to the pith [3,4]. With the spread of this pathogen, the canker disease has become more widespread and difficult to control, especially in China [4,5]. Currently, studies about *C. chrysosperma* are focused on the taxonomy, genetic diversity, histopathology, and gene function survey [3,4,6,7], while the molecular mechanism studies were few reported. For example, the mitogen-activated protein kinase (CcPmk1) and oxalic acid metabolism were required for the growth and virulence of *C. chrysosperma* [5,8,9]. The virulence effector CcCAP1, a member of the CAP superfamily (cysteine-rich secretory protein, antigen 5, and pathogenesis-related 1), from *C. chrysosperma* mainly localized to plant nucleus to suppress plants immune responses [2]. Additionally, the transcription factor CcSge1, belonging to the Gti1/Pac2 family, was essential to the hyphal radial growth, conidiation, and fungal virulence. Intriguingly, both CcSge1 and CcPmk1 positively regulated the expression of the putative effector gene *CcCAP1* in *C. chrysosperma* [10]. However, the molecular interaction mechanism between *C. chrysosperma* and plants is still unclear.

Plants and phytopathogens have coevolved for a long time and have established a two-layer interaction system: pathogen-associated molecular patterns (PAMPs)-triggered immunity (PTI) and effector-triggered immunity (ETI) [11,12,13]. Plant plasma membrane-localized pattern recognition receptors (PRRs) can sense pathogens through recognition of the highly conserved PAMPs, such as flagellin, chitin, and glucans, to activate the PTI [14,15]. Additionally, plant intracellular nucleotide-binding and leucine-rich repeat receptors (NLRs) can detect cytoplasmic effectors to induce the ETI responses, including the activation of a series of downstream defense-related responses [16,17,18,19].

Recently, studies have shown that ETI can enhance the PTI defense responses [20]. Phytopathogens secrete a series of effectors to disturb the plant defense responses and promote colonization, while some of them can trigger plant defense responses, known as avirulent protein. More and more evidence has demonstrated that effectors could locate to the plant nucleus to carry out their functions. HopA1_Pss61_ from *Pseudomonas syringae* is co-localized with *Arabidopsis* R protein RPS6 in the nucleus and cytoplasm, but the nucleus localization of HopA1_Pss61_ is required to induce cell death [21]. Pop2, an avirulence type III effector from *Raistonia solanacearum*, can be recognized by the RRS1-R in the nucleus, which will grant broad-spectrum resistance to several different strains of *Raistonia solanacearum*. The *Verticillium-*specific secreted protein VdSCP7 localized to the plant nucleus to trigger plant immunity, and its nuclear localization was required to activate plant defense responses [22]. Similar results were also reported in other effectors from different phytopathogens. In *Phytophthora infestans*, RXLR effector Pi04314 could be active in the host nucleus to reduce the expression of jasmonic and salicylic acid-related defense genes and induce the relocation of three host protein phosphatase 1 catalytic isoforms from the nucleolus to nucleoplasm during infection to enhance leaf colonization [23]. PvAVH53 located in the plant nucleus and could interact with tobacco importin-α genes, *NbImp-α1* and *NbImp-α2*, which were essential to induce cell death in *N. benthamiana* leaves. The silencing of importin-αs genes resulted in increased susceptibility to the oomycete pathogen *Phytophthora capsici* in *N. benthamiana* [24].

In this study, two candidate effectors (*CcSp31* and *CcSp84*), both containing a nuclear localization signal motif at the C-terminal, were functionally characterized in *C. chrysosperma*. The results showed that CcSp84 was required for the fungal pathogenicity and could trigger plant cell death in *N. benthamiana* leaves, while *CcSp31* deletion mutants displayed no obvious defects compared to the wild type, and it could not trigger cell death or suppress the INF1-induced cell death in *N. benthamiana*. Further analyses suggest that the plant nucleus localization of CcSp84 was important for its activation functions.

## 2. Results

### 2.1. Two NLS Motif Containing Candidate Effectors Were Induced during Infection Processes

**Figure 1:**
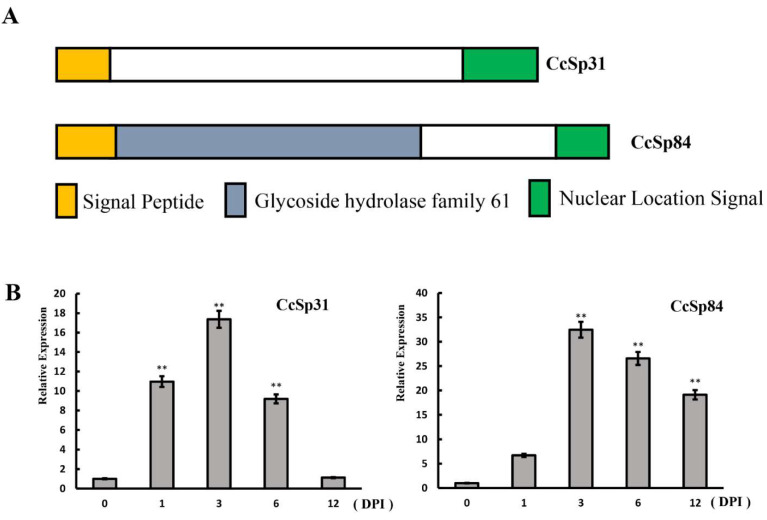
*CcSp31* and *CcSp84* were significantly up-regulated during infection stages. (**A**) Schematic diagram of candidate effectors *CcSp31* and *CcSp84*. (**B**) Relative expression levels of *CcSp31* and *CcSp84* during infection processes. The expression levels were monitored using reverse transcription-quantitative polymerase chain reaction (qRT-PCR). RNA was extracted at 0, 1, 3, 6, and 12 days post-inoculation (DPI) on the poplar twigs with *Cytospora chrysosperma.* The experiments were performed in triplicate. ** *p* < 0.01.

Pathogens secrete a series of effectors into the host cytoplasm and apoplast to disturb the host immunity [25]. As expected, numerous effector candidates were predicted in *C. chrysosperma* according to typical criteria, such as small in length, containing a signal peptide, no other transmembrane domains, and being rich in cysteine residues [26,27] (data not shown). Among them, two candidate effector genes, both containing a nuclear localization signal at the C-terminal, were selected, named *CcSp31* and *CcSp84*, in this study. *CcSp84* contained a glycoside hydrolase 61 domain, while no annotated domain was found in *CcSp31* (Figure 1A, Table 1). Remarkably, both of them were significantly up-regulated during the infection stages, and they both showed the highest expression levels at 3 dpi (Figure 1B), indicating their involvements in the fungal pathogenicity.

### 2.2. CcSp84 Induced Cell Death in N. benthamiana Leaves

**Figure 2:**
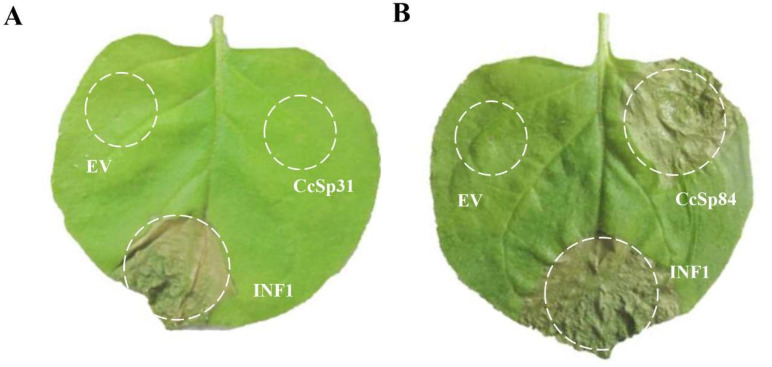
*CcSp31* and *CcSp84* were transiently expressed into *N. benthamiana* leaves by agro-infiltration. (**A**) pGR106 (Empty vector, EV), *CcSp31*-pGR106, and INF1-pGR106 were infiltrated in *N. benthamiana* leaves, respectively. (**B**) EV, *CcSp84*-pGR106, and INF1-pGR106 were infiltrated in *N. benthamiana* leaves, respectively. The pictures were taken at 3 dpi. The data represented have three independent biological repeats.

To investigate the function of *CcSp31* and *CcSp84* during the interaction processes, *CcSp31* and *CcSp84* were cloned into the binary vector pGR106 without signal peptide, respectively [28], and then transiently expressed in *N. benthamiana* leaves by using the *Agrobacterium tumefaciens*-mediated system. After 3 days of infiltration, *CcSp31* neither induced cell death nor suppressed the INF1-induced cell death in *N. benthamiana* leaves (Figure 2A and Figure S1). Remarkably, *CcSp84* could induce cell death in *N. benthamiana* leaves as the INF1 did, but it could not suppress the INF1-induced cell death in *N. benthamiana* leaves (Figure 2B), indicating that *CcSp84* acted as an elicitor and could induce cell death in *N. benthamiana* leaves.

### 2.3. CcSp31 and CcSp84 Were Not Required for Fungal Growth and the Resistance to H_2_O_2_

**Figure 3:**
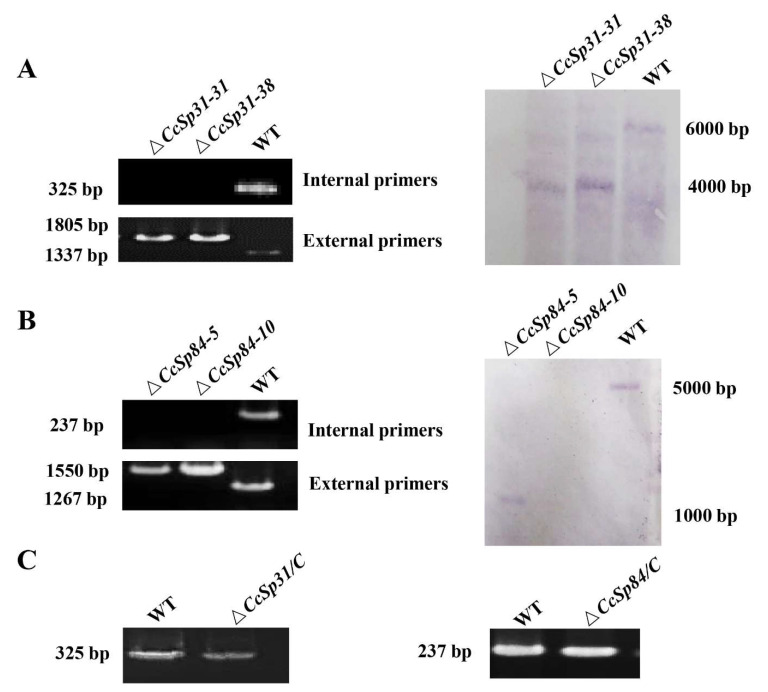
Screening for the *CcSp31* and *CcSp84* deletion mutants. (**A**,**B**) The *CcSp31* and *CcSp84* deletion mutants were confirmed by PCR with internal primers and external primers and Southern blot analysis. (**C**) The complementary strains were verified by PCR with internal primers.

**Figure 4:**
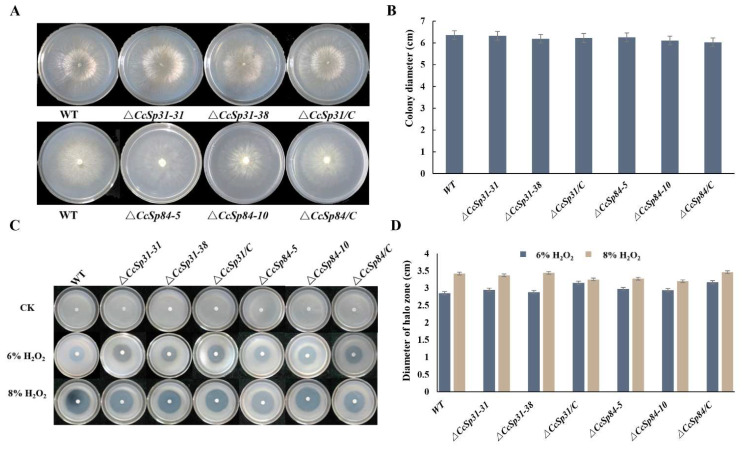
*CcSp31* and *CcSp84* were not required for the fungal growth and resistance to H_2_O_2_. (**A**) Colony morphologies of wild-type (WT), *CcSp31* and *CcSp84* deletion mutants, and complemented strains after 48 h of growth on PDA plates. (**B**) Colony diameters of the strains on PDA plates. (**C**) Colony morphology of wild-type, *CcSp31* and *CcSp84* deletion mutants, and each complemented strains treated with different concentrations of H_2_O_2_. The pictures were taken at 60 hpi. (**D**) Diameter of halo zones of the strains on PDA plates supplemented with H_2_O_2_.

To further determine the potential function of *CcSp31* and *CcSp84* in *C. chrysosperma*, the gene deletion fragments were constructed through the split-marker method (Supplementary Figure S2). Two deletion mutants of *CcSp31* (Δ*CcSp31-31* and Δ*CcSp31-38*) and two deletion mutants of *CcSp84* (Δ*CcSp84-5* and Δ*CcSp84-10*) were successfully obtained by using the PCR screening and Southern blot analyses (Figure 3A–C). The complementary strains were generated in a similar way (Supplementary Figure S1), named Δ*CcSp31/C* and Δ*CcSp84/C*. The mycelial plugs of normal wild type (WT), Δ*CcSp31-31*, Δ*CcSp31-38*, Δ*CcSp84-5*, Δ*CcSp84-10,* and their complementary strains were inoculated in the PDA medium. The results showed that no significant differences were found among all the strains (Figure 4A,B), indicating that *CcSp31* and *CcSp84* were not required for the fungal growth.

To examine the resistance of *CcSp31* and *CcSp84* deletion mutants to H_2_O_2_, conidial suspensions of WT, *ΔCcSp31,* *ΔCcSp84,* *ΔCcSp31/C,* and *ΔCcSp84/C* (1 × 10^6^ conidiospore/mL) were added into homogeneously melted and warmed PDA medium, then a filter paper with 6% and 8% H_2_O_2_ was placed in the middle of the plates, as described previously (Wang et al., 2018). No distinguish differences in responses to the H_2_O_2_ stress were observed among the *CcSp31* and *CcSp84* deletion mutants, complementary strains, and the WT (Figure 4C,D). The data illustrate that *CcSp31* and *CcSp84* were dispensable for fungal growth and the resistance to H_2_O_2_.

### 2.4. CcSp84 Was Essential for Fungal Pathogenicity

**Figure 5:**
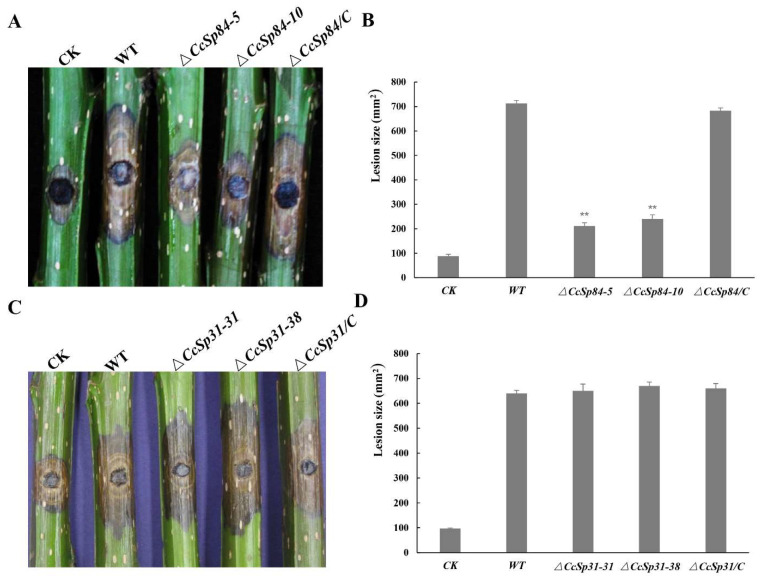
The *CcSp84* deletion mutants reduce the pathogenicity of *C. chrysosperma.* (**A**,**B**) Inoculated poplar branches with wild-type, *CcSp84* deletion mutants, and complemented strains, the lesion areas were measured at 4 dpi. (**C**,**D**) Inoculated poplar branches with wild-type, *CcSp31* deletion mutants, and complemented strains, the lesion areas were measured at 4 dpi. The asterisks on the bars indicate a significant difference from the wild-type strain (** *p* < 0.01). The experiments were repeated three times.

To calculate the putative roles of *CcSp31* and *CcSp84* in the fungal pathogenicity, we scalded the *Populus euramericana* branches and then inoculated them with mycelial plugs of WT, Δ*CcSp31* and Δ*CcSp84,* and their complementary strains. As shown in Figure 5A, B, the lesion areas were significantly reduced in Δ*CcSp84-5,* and Δ*CcSp84-10* inoculated branches, compared to that in the WT and complementary strains, while comparable lesion areas were observed in *CcSp31* deletion mutants and WT inoculated branches (Figure 5C,D). The above results suggest that CcSp84 is a pathogenicity-related elicitor in *C. chrysosperma*; therefore, *CcSp84* was selected for further analyses.

### 2.5. The Nuclear Localization of CcSp84 Was Required to Induce Plant Defense Responses

**Figure 6:**
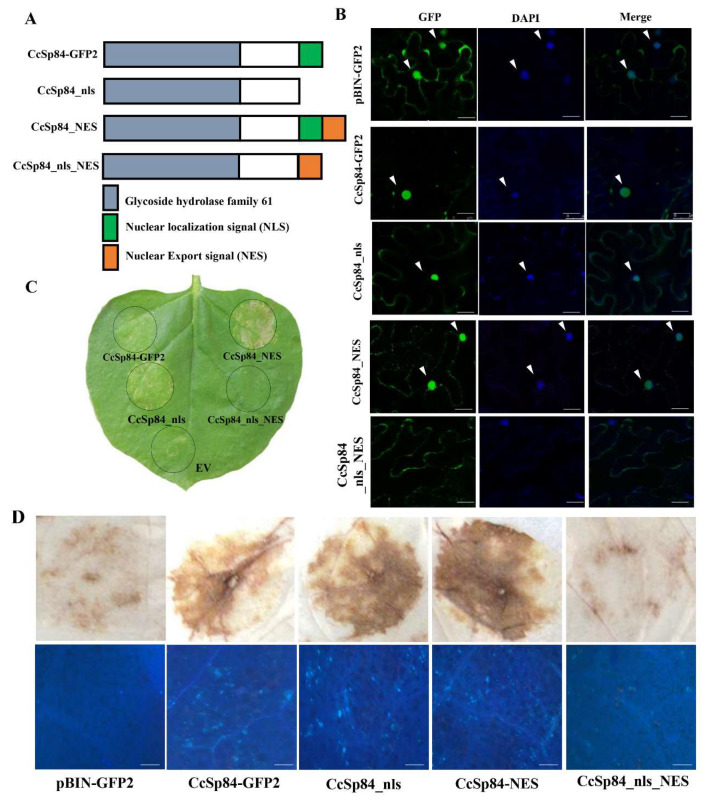
*CcSp84* located in the plant nucleus to trigger plant defense responses. (**A**) Diagram structure of different mutant types of *CcSp84* fused to GFP. (**B**) Subcellular localization of GFP-tagged *CcSp84* mutant types when transiently expressed in *N. benthamiana*. Confocal Microscope SP8 with wavelengths of 488 and 406 nm for GFP and DAPI, respectively. The pictures were photographed at 3 dpi. Bars, 5 μm. (**C**) Different mutant types of *CcSp84* were transiently expressed in *N. benthamiana*. (**D**) The detection of ROS and callose deposition in *N. benthamiana* leaves at 24 h post-inoculation. Bars, 200 μm.

As shown in Figure 1A, *CcSp84* contains a predicted NLS motif at C-terminal. In order to clarify the subcellular localization of *CcSp84* in the plant cell and the functions of the NLS motif, four recombinant plasmids were constructed, including *CcSp84-GFP2*, *CcSp84_nls* (deletion of NLS sequences), *CcSp84_NES* (adding the NES sequences at the C-terminal), and *CcSp84_nls_NES* (deletion of NLS sequences and adding the NES sequences at the C-terminal) (Figure 6A). Then, these recombinant plasmids of *CcSp84* were introduced into the *A. tumefaciens* GV3101 and transiently expressed in *N. benthamiana* leaves. Confocal microscopy results showed that the fluorescence of *CcSp84-GFP* was mainly localized in the plant nucleus, while the fluorescence signal of *CcSp84_nls* and *CcSp84_NES* could be observed in both plant nucleus and cytoplasm. Additionally, the fluorescence signal of *CcSp84_nls_NES* was only detectable in the plant cytoplasm but not the plant nucleus (Figure 6B). These results indicated that *CcSp84* was mainly located in the plant nucleus and the NLS motif was important for its plant nucleus localization.

To analyze the relationship between the *CcSp84* localization and its function, the response of *N. benthamiana* leaves infiltrated the recombinant *A. tumefaciens* containing different mutants of *CcSp84* plasmids was observed. The results showed that CcSp84_GFP2, CcSp84_nls, and CcSp84_NES could induce different levels of plant cell death after three days of inoculation, whereas *CcSp84_nls_NES* was unable to induce cell death (Figure 6C). Additionally, obvious ROS accumulation and callosum deposition were detected in the *N. benthamiana* leaves infiltrated with CcSp84_GFP2, CcSp84_nls, and CcSp84_NES at 24 h post-inoculation, while comparable results were found between control treatment and CcSp84_nls_NES (Figure 6D). Together, these results demonstrate that the nuclear localization of CcSp84 is essential to trigger plant immunity.

### 2.6. Expression of CcSp84 Could Induce the Expression of Defense-Related Genes in N. benthamiana

**Figure 7:**
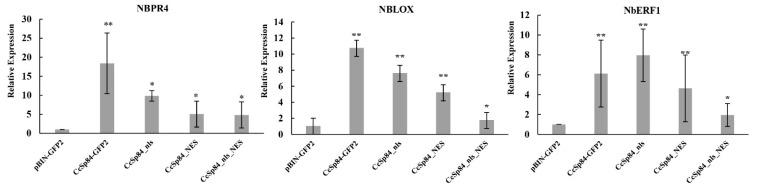
Relative expression levels of defense-related genes involved in JA and ET signal pathways in *N. benthamiana*. The marker genes of JA and ET-dependent defense responses were significantly up-regulated after being transiently expressed the CcSp84 in *N. benthamiana*. The experiments were performed three times. * *p* < 0.05, ** *p* < 0.01.

Previous studies have proved the synergism between jasmonic acid (JA) and ethylene (ET) signaling pathways, which are required for the resistance against necrotrophic pathogens through the regulation of plant defense-related genes [22,29,30]. To assess whether *CcSp84* could affect the expression of defense genes in the JA and ET signaling pathways, the expression of marker genes involved in JA and ET signaling pathways were calculated, including NbPR4 (Niben101Scf12045g06025.1), NbLOX (Niben101Scf01434g03006.1), and NbERF1 (Niben101Scf00454g04003.1) [31,32,33]. As shown in Figure 7, the expression of NbPR4, NbLOX, and NbERF1 was significantly induced in the *N. benthamiana* leaves transiently expressed *CcSp84*, *CcSp84_nls*, and *CcSp84_NES* recombinant strains compared with control, which indicated that transiently expressed CcSp84 in *N. benthamiana* would activate the JA and ET-related signal pathway to trigger plant immune responses.

## 3. Discussion

In plant–pathogen interactions, the plant nucleus is a core battlefield, and various interactions were involved. Effectors and receptors play an important role in determining the overall outcome of infection [22,33,34]. In this study, we identified and functionally characterized a small, cysteine-rich secreted protein, *CcSp84*, which acted as a key virulence-related candidate effector of *C. chrysosperma*. *CcSp84* was strongly induced during infection stages and was required for the pathogenicity of *C. chrysosperma*. Transient expression of *CcSp84* on the *N. benthamiana* leaves could trigger plant immune responses, and its nuclear localization was essential to activate the plant defense responses.

It is well known that phytopathogens secret a large number of effectors to the extracellular matrix during the infection process. Some of them are located at the extracellular matrix, while some of them are located at the cytoplasm [35,36]. The different locations of the effectors may represent various regulation manners. For example, VdEG1 and VdEG3 from *Verticillium dahliae*, which belonged to the Glycoside Hydrolase Family (GH12), were identified as apoplastic elicitors and mediated the plasma membrane-localized receptor BAK1 to regulate the host immunity [37]. PstGSRE1 from *Puccinia striiformis* f. sp. *tritici* (Pst) targeted the ROS-associated transcription factor TaLOL2 by disrupting the nuclear localization of TaLOL2 and suppressing the ROS-mediated cell death induced by TaLOL2 [38]. Additionally, a putative effector VdSCP7 from *Verticillium dahliae* contained an NLS motif, which was also located in the plant nucleus and modulated plant immunity. Here, CcSp84 contained a predicted NLS motif (Figure 1A) and was localized to the plant nucleus to induce plant immune responses (Figure 6C). The results suggest that the NLS motif may lead to the nucleus location of effectors in the plant cell. More importantly, many studies have reported that the correct localization of effectors to specific subcellular compartments is essential for their functions. For example, transiently expressed full-length PsXEG1 (including signal peptide) or PR1-PsXEG1^20–241^, an apoplast effector from *Phytophthora sojae*, could trigger cell death, while transiently expressed PsXEG1^20–241^ (without signal peptide) did not trigger cell death in *N. benthamiana*, indicating that the PsXEG1 must target the apoplast to achieve its functions [39]. Similar results were also found in the XEG1 homologs from *Botrytis cinerea* and *V. dahliae* [37,40]. In this study, transiently expressed *CcSp84* in *N. benthamiana* leaves could induce cell death, and fluorescence observation found that *CcSp84* is located in the plant nucleus. Additionally, *CcSp84_NES* was localized to the plant cytoplasm and nucleus, indicating that the nuclear export signal (NES) sequence can partly induce the exportation of *CcSp84* from the nucleus to cytoplasm. However, when deleting the NLS sequence and adding an NES sequence to the *CcSp84*, the GFP signal was almost abolished in the nuclei in *CcSp84_nls_NES* expressing leaves. Similar results were also found in the plant nuclear localization effector VdSCP7 from *Verticillium dahliae*, which also contains an NLS sequence [22]. Therefore, we speculated that when the protein contains both NLS and NES sequences, its localization may depend on the functional ability of these two motifs. Intriguingly, its elicitor activity was compromised when it manually exported *CcSp84* to the cytoplasm region, indicating that the plant nuclear localization is essential for *CcSp84* to trigger plant immune responses.

Generally, the expression of secreted effectors would be induced during the infection or colonization processes, and several of them may be stage-specific [41]. Additionally, numerous effectors were reported to be involved in pathogenicity because of their crucial roles during the interactions between pathogens and host plants [13,36,42,43]. In stripe rust fungus, a haustorium-specific effector, Pst_12806, was significantly up-regulated during infection stages, and it could suppress the plant basal immunity by reducing the callose deposition and the expression of defense-related genes [44]. However, many of them did not show obvious defects in pathogenicity with single deletion of the effector genes, which was possibly due to the functional redundancy. For example, the LysM-type effector from *Zymoseptoria tritici*, Mgx1LysM, was essential for the lesion’s extension through binding to the chitin and then suppressed the chitin-induced ROS burst, and was able to protect fungal hyphae against host chitinase hydrolysis [45]. In this study, *CcSp84* was significantly induced during the infection stages, and transient expression of *CcSp84* in *N. benthamiana* would cause cell death and activate the plant defense responses, including the burst of ROS, deposits of callosum, and induced expression of defense-related genes. Importantly, *CcSp84* was required for fungal pathogenicity. On the other hand, *CcSp31* was also significantly up-regulated during the infection stages, but the deletion of *CcSp31* did not exhibit defects in fungal pathogenicity compared to the wild type. Therefore, we speculated that the functional redundancy of effectors and the dependence of expression pattern during infection stages may lead to the non-visualization of fungal virulence.

Increasing evidence is available for the positive roles of plant JA-mediated signaling against biotrophic and necrotrophic pathogens [30,46,47,48]. *Pseudomonas syringae* releases type III effector HopX1, a cysteine protease, which has been reported to interact with the ZIM domain of jasmonate family members and degrade them, resulting in plant susceptibility [49]. The AvrB effector RIN4 activated the plasma membrane ATPase AHA1 to cause an alteration in membrane potential, which increased the interaction between COI1 and JAZ, ultimately leading to degradation of the JA-related proteins [50]. Here, we investigated the expression pattern of three marker genes related to the immune-related signaling pathways: JA and ET, and the results demonstrated that *CcSp84* could positively regulate the JA and ET signaling pathway.

Collectively, we functionally characterized two putative NLS motifs containing effectors, *CcSp31* and *CcSp84,* from *C. chrysosperma*. The *CcSp31* deletion mutants displayed no defects in the vegetative growth, stress response, and virulence compared to the wild-type, while *CcSp84* was critical for fungal pathogenicity, which could also induce cell death and immune responses in *N. benthamiana*. Further analysis revealed that the plant nucleus localization of *CcSp84* was essential to trigger the plant defense responses.

## 4. Material and Methods

### 4.1. Fungal Strains and Plants Growth Conditions

The wild-type (WT) strain (CFCC_89981) of *C. chrysosperma* was preserved in the forest pathology laboratory in Beijing Forestry University (Strain No: G-YS-11-C1). The WT, deletion, and complementation mutants were all cultured in a potato dextrose agar (PDA) medium (200 g potato filtrate, 20 g glucose, 15 g agar per liter) at 25 °C in the dark. Conidia were incubated in a yeast extract peptone dextrose (YEPD) medium (3 g yeast extract, 10 g bacto peptone, and 20 g glucose per liter) for the preparation of protoplasts and transformants. Furthermore, TB_3_ medium (3 g yeast extract, 3 g casamino acids, 200 g sucrose, 7 g agar per liter) with 30 μg/mL hygromycin, was used to select transformants. The cultures of *C. chrysosperma* were grown in a liquid potato dextrose broth (PDB) medium (200 g potato filtrate, 20 g glucose per liter) at 25 °C, shaking at 250 rpm for DNA or RNA extraction. *E. coli* DH5α cultured in lysogeny broth (LB) at 37 °C were used for plasmid construction. *Agrobacterium* strain GV3101 was cultured at 30 °C and used for *Agrobacterium*-mediated transient expression in *N. benthamiana* leaves. *N. benthamiana* was grown in a greenhouse at 25 °C and 70% relative humidity with a 16/8 h of day/night light for about four weeks.

### 4.2. Bioinformatics Analysis

The putative effectors of *C. chrysosperma* could be identified by several prominent features, such as the signal peptide, cysteine-rich domain, small length, and no additional transmembrane structure [51]. In this research, the SignalP 5.0 Server (http://www.cbs.dtu.dk/services/SignalP-5.0/, accessed on 27 August 2019) and TMHMM Server 2.0 (http://www.cbs.dtu.dk/services/TMHMM/, accessed on 27 August 2019) were used to search the signal peptide and the domain of transmembrane structure. The Interpro (http://www.ebi.ac.uk/interpro/, accessed on 28 August 2019), Smart Page (http://smart.embl-heidelberg.de/, accessed on 28 August 2019) and cNLS Mapper (http://nls-mapper.iab.keio.ac.jp/cgi-bin/NLS_Mapper_form.cgi, accessed on 28 August 2019) were used to detect the gene family, functional domains, and nuclear localization signal motif.

### 4.3. Gene Knockout and Complementation

The target genes, *CcSp31* and *CcSp84*, were knocked out by the split-marker method [52,53]. According to the method, the upstream and downstream flanking sequences, about 1.5 kb of *CcSp84,* were amplified by the polymerase chain reaction (PCR) with primer pairs CcSp84-5F-F/CcSp84-5F-R and CcSp84-3F-F/CcSp84-3F-R (Supplementary Table S1), respectively. The upstream and downstream flanking sequences of *CcSp84* were fused with hygromycin B resistance gene by using the overlapped PCR with the primer pairs *CcSp84-5F-F*/Hygromycin-R or Hygromycin-F/*CcSp84-3F-R* (Supplementary Table S1). All the sequences of fragments were confirmed by sequencing analysis. Then the recombinant fragments were co-transformed into protoplasts of WT strain through PEG-mediated transformation. The transformed strains were cultured in the TB3 medium with 30 μg/mL hygromycin and then were selected and verified by the PCR with the primer pair External-CcSp84-F/R and Internal-CcPs84-F/R. The Southern blot analysis was performed to confirm the single copy homologous recombination event by using the DIG High Prime DNA Labeling and Detection Starter Kit I, following the manufacturer’s protocol (Roche, Germany). The genomic DNA from the WT strain, *CcSp31* and *CcSp84* deletion mutants, were digested by restriction enzyme *Nco*I and then separated by gel electrophoresis. The probe fragments for *CcSp31* and *CcSp84* were acquired by PCR amplification with the primers CcSp31-Pro-F and CcSp31-Pro-R or CcSp84-Pro-F and CcSp84-Pro-R, respectively, and then labeled with DIG.

For complementation, the full length of *CcSp84*, including the 1.5 kb native promoter, *CcSp84* genomic sequences, and 0.2 kb terminal sequences and geneticin-resistant cassettes, were co-transformed into the protoplast of the deletion mutant. Then, transformants were selected by 30 μg/mL hygromycin and 50 μg /mL geneticin. The successful complementary strain was named as Δ*CcSp84/C* in this study. A similar strategy was used for the deletion and complementation of *CcSp31*.

### 4.4. Plasmid Construction and Transient Expression

To determine the eliciting or inhibitory activity of candidate effectors, the coding sequence of *CcSp84* without signal peptide (mature type) was amplified from the cDNA library of *C. chrysosperma* with primer pairs CcSp84-sp-F/CcSp84-R. The plasmid pGR106 was linearized by Cla I/Not I and then ligation with the purified fragment of *CcSp84* to obtain the CcSp84-pGR106 recombinant plasmid. The CcSp31-pGR106 recombinant plasmid was constructed in the same way with primer pairs CcSp31-sp-F/CcSp84-R.

In order to verify the influence of different positioning conditions on CcSp84, several mutant types of CcSp84 were generated and cloned into pBINGFP2, including the *CcSp84_nls_NES* (the NLS sequence was removed, and a nuclear export signal (NES) sequence was added at the C-terminal), *CcSp84_nls* (the NLS sequence in *CcSp84* was removed), and *CcSp84_NES* (an NES sequence was added at the C-terminal of *CcSp84*). The original plasmids pGR106 (EV) and pBINGFP2 were kindly provided by Pro. Daolong Dou from Nanjing Agricultural University.

All the recombinant plasmids were transformed into *Agrobacterium tumefaciens* strain GV3101 by 2500 volts electric shocking [54]. The recombinant strains were cultured in LB liquid medium with 50 μg/mL kanamycin and rifampin resistance at 200 rpm and 2 °C for 48 h, and then the strain’s solution was washed with the 10 mM MgCl_2_ solution three times, resuspended with MMA buffer (10 mM MgCl_2_, 10 mM 2-ethanesulfonic acid, and 200 μM acetosyringone), and cultured for 4 h. The culture solution was then diluted by adjusting the OD_600_ to 0.4 with MMA buffer and infiltrated into the leaves of *N. benthamiana* with a 1-mL syringe.

### 4.5. Confocal Fluorescent Analysis

After 2 days of agro-infiltration of *A. tumefaciens* GV3101 containing pBINGFP2, CcSp84-pBINGFP2, CcSp84_nls-pBINGFP2, CcSp84_NES-pBINGFP2, and CcSp84_nls_NES-pBINGFP2, the leaves of *N. benthamiana* were collected and infiltrated with 5 mg/mL DAPI (4′6-diamidino-2-phenylindole). After 1 h, the leaves were cut into 8 × 8 mm pieces and observed by the Laser confocal microscope (Leica SP8, Weztlar, Germany). The GFP fluorescence was excited using 488 nm laser line.

### 4.6. Detection of Reactive Oxygen and Callosum

To detect the ROS burst, the *N. benthamiana* leaves were stained and shaken in 1 mg/mL 3, 3’-Diaminobenzidine (DAB) solution (Sigma, USA) for 2 h after 24 h post-inoculation. Then, the leaves were discolored with 95% ethanol and observed by the microscope (Leica DM 2500, Weztlar, Germany). Callose deposition was detected by using aniline blue staining [55]. Agro-infiltrated *N. benthamiana* leaves were de-stained with 95% ethanol and then stained with 0.05% aniline blue in 0.067 mol K_2_HPO_4_ buffer overnight and subsequently observed using a Leica DM2500 microscope (Leica, Weztlar, Germany).

### 4.7. Pathogenicity Assay

For the pathogenicity assay, branches of annual susceptible host *Populus euramericana* were cut to 15 cm long and sealed by the wax. Then, the branches were scald by a 5 mm-diameter hot soldering iron and inoculated with *C. chrysosperma* mycelial plugs. After inoculation, branches were preserved in 25 °C and a wet environment. The lesions were photographed and measured at 4 dpi. The experiment was repeated three times.

### 4.8. RNA Extraction and qRT-PCR Analysis

The samples of *C. chrysosperma* were collected at 48 h post culture in PDB, and *N. benthamiana* leaves infiltrated with *A. tumefaciens* GV3101 containing different recombinant plasmids were collected 24 h post-infiltration. Total RNA was extracted with RNA Easy Fast Plant Tissue Kit (TIANGEN) according to the manufacturer’s instructions. First-strand cDNA was synthesized with 2 ug RNA by ABScript II cDNA First-stand Synthesis Kit (ABclonal). The following qPCR used the qPCR SYBR Green Master Mix (Yeasen). The NbActin was set as endogenous control, the relative expression levels were calculated by the 2 −ΔΔCt method with three independent biological replicates. All primers used in this study were listed in Supplementary Table S1.

## Figures and Tables

**Table 1:** Basic information of selected candidate effectors CcSp31 and CcSp84.

Gene Name	*CcSp31*	*CcSp84*
Gene ID	GME4592_g	GME8128_g
Signal Peptide	Y	Y
Protein length (SP truncated)	277 (260)	315 (293)
Cysteines number	5	3
homologous gene	Conserved in fungi	Conserved in fungi
NLS sequence	KKMRKRHSDNGVRMPWKKVKR	PSKCKKRRHARD

## Data Availability

Data are contained within the article or Supplementary Material.

## Supplementary Materials

The following are available online at https://www.mdpi.com/article/10.3390/ijms23031614/s1.
