# Supplementary material for: A Putative Effector CcSp84 of Cytospora chrysosperma Localizes to the Plant Nucleus to Trigger Plant Immunity
Source: Int J Mol Sci. 2022 Jan 30;23(3):1614. doi: 10.3390/ijms23031614 (PMC8835870; doi:10.3390/ijms23031614)
Supplement: Supplementary file 1 [file ijms-23-01614-s001.zip › ijms-1555806-Figures S1 and S2.pdf]

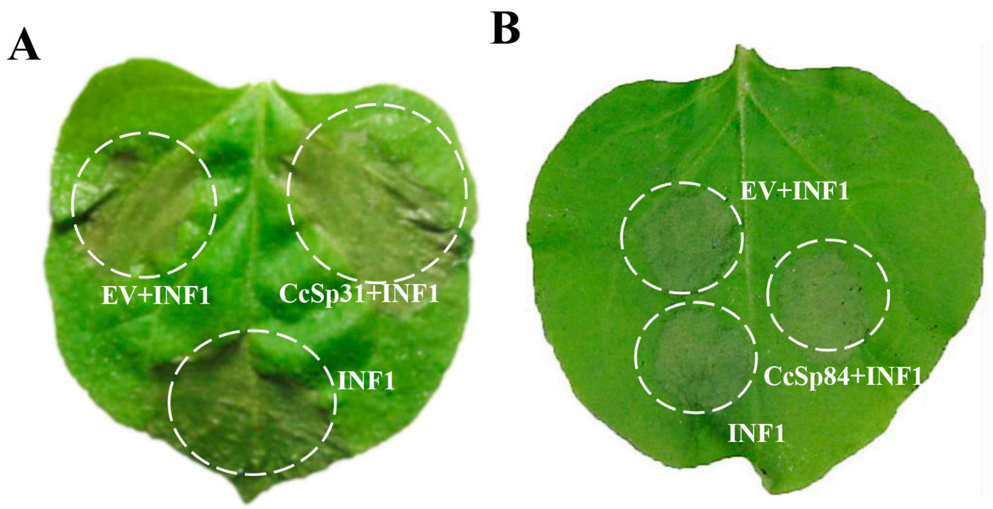

Figure S1. Transient expression of CcSp31 and CcSp84 in *N. benthamiana* leaves. (A) pGR106-EV and pGR106-CcSp31 were co-inoculated with INF1 in *N. benthamiana* leaves, pGR106-INF1 was set as positive control. (B) pGR106-EV and pGR106-CcSp84 were co-inoculated with INF1 in *N. benthamiana* leaves. Pictures were taken at 3 dpi.

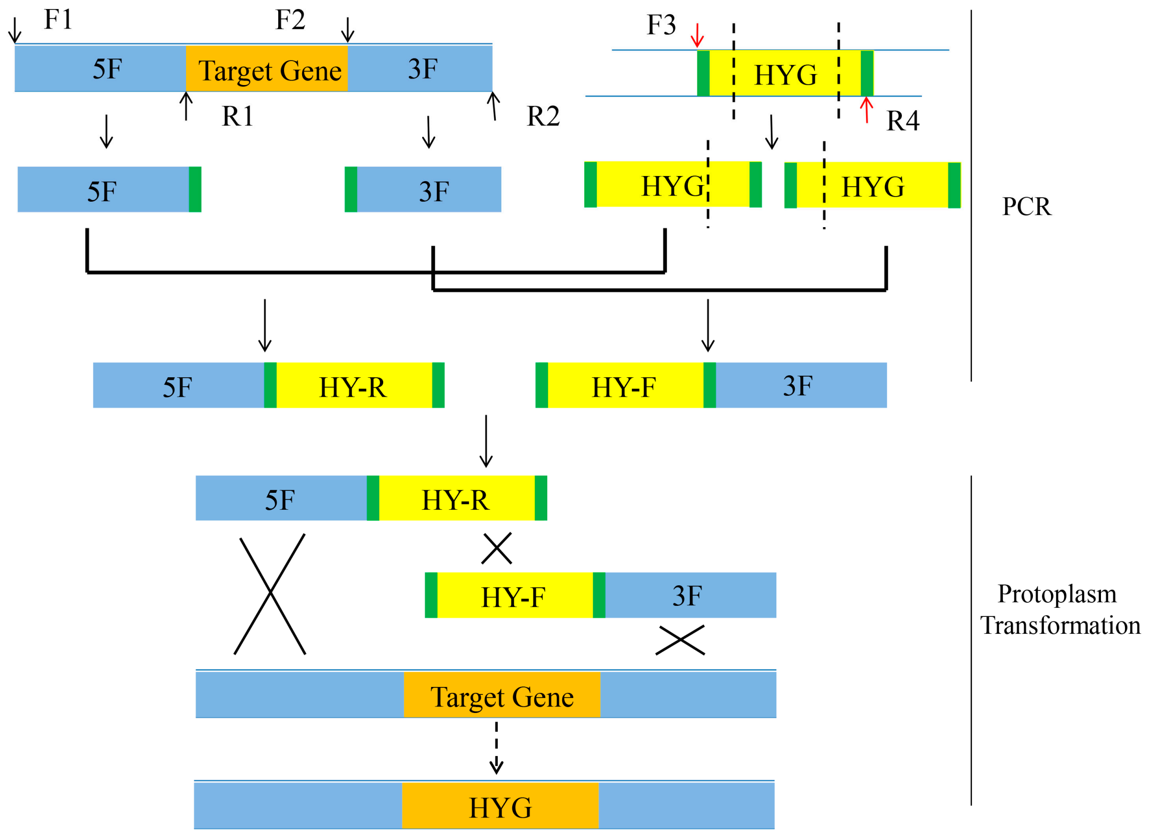

Figure S2. Strategy of gene deletion. Split marker was the method that used to replace CcSp84 fragment with hygromycin cassette. .
